# Does a Single Exposure to General Anesthesia Have a Cumulative Effect on the Developing Brain after Mild Perinatal Asphyxia?

**DOI:** 10.3390/life12101568

**Published:** 2022-10-09

**Authors:** Sebastian Isac, Bogdan Pavel, Maria Dobre, Elena Milanesi, Irina-Mihaela Matache, Raul-Mihai Paun, Artsiom Klimko, Mara Ioana Iesanu, Gabriela Droc, Ana-Maria Zagrean

**Affiliations:** 1 Department of Physiology, Faculty of Medicine, Carol Davila University of Medicine and Pharmacy, 020021 Bucharest, Romania; 2 Department of Anesthesiology and Intensive Care I, ‘Fundeni’ Clinical Institute, 022328 Bucharest, Romania; 3 Victor Babes National Institute of Pathology, 050096 Bucharest, Romania; 4 Laboratory of Molecular Neuro-Oncology, Department of Neurology, University Hospital Zurich, 8091 Zürich, Switzerland

**Keywords:** perinatal asphyxia, general anesthesia, neuroinflammation, glial activation, behavioral impairments, hippocampus

## Abstract

Background: General anesthesia (GA) in pediatric patients represents a clinical routine. Factors such as increased birth age and maternal chronic conditions cause more infants to experience hypoxic-ischemic encephalopathy, an additional risk for anesthesia. Aim: This study evaluates the effect of one sevoflurane-induced GA episode on the immature brain previously exposed to perinatal asphyxia (PA). Methods: Postnatal day 6 (PND6) Wistar rats were exposed to a 90-min episode of normoxia/PA and at PND15 to a 120-min episode of normoxia/GA. Four groups were analyzed: Control (C), PA, GA, and PA-GA. Post-exposures, fifteen pups/group were sacrificed and the hippocampi were isolated to assess S-100B and IL-1B protein levels, using ELISA. At maturity, the behavior was assessed by: forced swimming test (FST), and novel object recognition test. Results: Hippocampal S-100B level was increased in PA, GA, and PA-GA groups, while IL-1B was increased in PA, but decreased in PA-GA. The immobility time was increased in PA and PA-GA, in FST. Conclusions: Both PA and GA contribute to glial activation, however with no cumulative effect. Moreover, PA reduces the rats’ mobility, irrespective of GA exposure, while memory evaluated by the novel object recognition test was not influenced.

## 1. Introduction

In the actual medical practice, numerous pediatric patients have a high addressability to various surgical fields, each mandating appropriate anesthesia. The continuous scientific progress led to the discovery of new agents capable of faster induction and quicker recovery, their potential toxic side effects remaining unknown, especially in the immature brain. Even less data is available regarding children’s exposure to general anesthesia (GA) in patients exposed to perinatal asphyxia (PA). While in a period of rapid neurodevelopment, children are more vulnerable to neurotoxic substances compared to adults [1].

Due to the increased addressability of pediatric patients to various surgical fields, appropriate anesthesia becomes an unavoidable need. Despite their widespread use, little data is available in the current medical literature to quantify the vulnerability of a young developing brain exposed to anesthetics and potential neurotoxic substances. Considering this deleterious potential, it becomes imperative to analyze the cumulative effect of anesthesia and PA in the same target population. Data accumulated over the last two decades led to the conclusion that the hippocampus is, due to its selective vulnerability to injury and oxidative stress, a reference point in the study of neurologic effects of different substances; therefore, we assessed the lesion and neuroinflammation at this level [2,3,4].

General anesthetics alleviate distress and exaggerated hemodynamic and nervous responses to pain and stressful stimulation, allowing surgery and diagnostic procedures to occur in optimal conditions. The exact mechanisms of hypnotics’ action are complex but incompletely described in the literature, with no particular evidence of their influence on the developing brain [5]. Most hypnotics act by facilitating the inhibitory neural pathways (agonists of gamma amino butyric acid A (GABA-A) or benzodiazepine receptors), leading to loss of consciousness, thus enabling the surgical procedure without awareness. During the early stages of development, the GABAergic synapse has an excitatory potential [6,7,8], leading to disturbances in neural homeostasis secondary to anesthesia. As an exception, ketamine acts as a non-competitive NMDA (N-methyl-D-aspartate) receptor antagonist leading to a state of hyperexcitation called “dissociative anesthesia”. NMDA receptors are important molecular tools in synaptic plasticity and learning and memory functions [9], and their blockage could have a neurotoxic potential on pediatric patients and should be used more cautiously.

It is known that GA affects neural homeostasis in a complex and heterogeneous way, but its potential harmful effect, as an independent risk factor on the immature brain, is still an unresolved issue. Emerging studies, mainly carried out in early postnatal animals, have demonstrated evidence of neurotoxicity from both inhalational and intravenous anesthetics, materialized through widespread neurolysis, alteration in neuronal circuitry, and long-term neurological disabilities [10]. However, the applicability of animal data to anesthesia practice remains uncertain [11]. Retrospective observational human studies have suggested a dose-dependent association between multiple anesthetic exposures in early childhood and subsequent learning disability leading to maladaptive behaviors [12]. Another topic of much debate is the complexity and nuances of different anesthetic methods, patients, and procedures. Studies provide a direct relationship between anesthesia quality and its neurotoxic effect, rather than anesthesia type or exposures frequency [13].

Regarding the anatomical structures that might be vulnerable to anesthesia, the hippocampus has three essential cognitive functions: response inhibition, episodic memory, and spatial cognition. Given its impressive neuroplasticity capacity, the hippocampus is one of the most vulnerable areas in the immature brain. Previous studies on humans and animals showed a neurotoxic effect of anesthetics on the immature hippocampus [14,15].

PA increases hippocampal inflammation and injury, making this structure a reference point in the study of potential neurodevelopmental risks associated with pediatric anesthesia [16].

One unanimous consensus in today’s medical world is that additional preclinical and clinical research efforts are urgently required to address this concern for child health. The present literature reports short-term effects in children, neglecting the possible long-term neuropsychiatric outcomes. Our study aims to investigate the role of one brief GA episode in early childhood on the developing brain previously exposed to a mild PA exposure. To this aim, we assessed the post-anesthesia hippocampal neuroinflammation and injury by measuring the levels of interleukin 1B (IL-1B) and S-100B protein, respectively. Also, at the adulthood stage, we evaluated the locomotor ability, short and long-term memory, anxiety level and depressive-like behavior.

## 2. Materials and Methods

All exposures were conducted on Wistar rats, with the approval of the local ethics committee for animal research under the European Communities Council Directive 86/609/EEC on the protection of animals used for scientific purposes (No. 3974/15.02.2022). Efforts were made to reduce the number of animals used and their suffering while maintaining statistical power. The pups were housed in standardized conditions: 12-h day-night cycles, a constant ambient temperature of 21 °C, and ad libitum access to food and water. The animal procedures were carried out respecting the 3Rs principle (Replace, Reduce, Refine). Welfare assessment focused on the animal’s appearance, weight, and behavior during all experimental steps.

**Figure 1:**
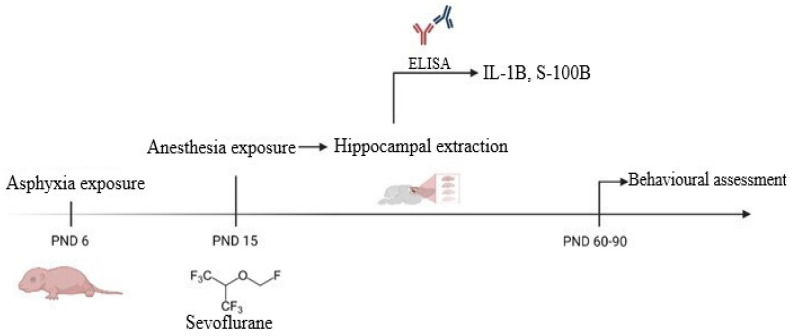
Experimental setup. PND—postnatal day; IL-1B—interleukin 1B; S-100B—S-100B protein.

On postnatal day (PND) six, 88 pups were randomly distributed into four experimental groups—control (C), perinatal asphyxia (PA), general anesthesia (GA), perinatal asphyxia, and anesthesia (PA-GA). From these, 15 pups/group, male and female, were used for ELISA assessment and 7 pups/group, male-only, were used for behavioral tests. The experimental setup is summarized in Figure 1.

### 2.1. Asphyxia Exposure

The asphyxia exposure was performed at PND6 in a flow-through chamber system and consisted of a 90-min exposure to either normoxia or asphyxia using a hypoxic-hypercapnic gas mixture (9% O_2_, 20% CO_2_ in N_2_) at a constant rate of 2 L/min using an open non-rebreathing system. This model of PA was developed by Helmy et al. [17] and modified and previously used by us in several studies [16,18]. During the asphyxia episode, the ambient temperature was controlled using a heating pad to maintain the body temperature of the pups at 36.6 °C. Additionally, vital signs (temperature, heart rate, breath rate, breath distension, and arterial oxygen saturation) were noninvasively monitored throughout the exposure in one pup randomly chosen using the MouseOX Plus system (STARR Life Sciences Corp, Oakmont, PA, USA). After the asphyxia event, the pups were returned to their mothers. No pups died in consequence of asphyxia exposure.

### 2.2. General Anesthesia Exposure

On PND15, the pups were exposed to either normoxia (fresh room air) or anesthesia consisting of inhalation of sevoflurane (S.C. Rompharm Company S.R.L, Otopeni, Romania) exposure in 100% O_2_ for 120 min using an open anesthesia circuit. The body temperature of the pups was maintained at 36.6 °C using a heating pad. Anesthesia was induced with 5% sevoflurane at a flow of 6 L/min for 2 min and maintained using 3% sevoflurane at a flow of 2 L/min, considering a potential concentration of sevoflurane ranging between 2.4–2.6%, which is equivalent with 1 MAC (minimum alveolar concentration). After anesthesia exposure, the pups were either sacrificed for hippocampal extraction and downstream analysis or kept for later adulthood locomotor and behavioral assessment. Two pups died after the anesthesia exposure, probably due to a respiratory complication, considering that our protocol did not allowed any resuscitation or intervention in case of major complications.

### 2.3. Experimental Groups

Consecutive to exposures to asphyxia/normoxia (PND6) and anesthesia/normoxia (PND15), 4 experimental groups resulted (15 pups randomly distributed/group), as follows: group C (pups exposed just to normoxia), group PA (pups exposed to asphyxia and normoxia at PND15), group GA (pups exposed to normoxia at PND 6 and anesthesia in PND 15), and PA-GA (pups exposed to both asphyxia and anesthesia).

### 2.4. Hippocampal Neuroinflammation and Injury Assessment by ELISA

At PND15, part of the pups exposed to GA/normoxia (*n* = 15/group) was euthanized by cervical dislocation within the first 30 min post-exposure. Then, the hippocampi were dissected and isolated from the surrounding meningeal structures, rinsed with 4 °C phosphate-buffered saline (0.02 mol/L, pH = 7.0–7.2), minced, homogenized, sonicated for 10 min, and centrifuged at 5000× *g* for 5 min at 4 °C, according to manufacturer recommendation. The supernatant was stored at −20 °C until all samples were collected. The levels of hippocampal IL-1B and S-100B were assessed using the IL1-B and the S-100B ELISA kits (antibodies-online GmbH, Aachen, Germany). The total protein content measurement was assessed using the Pierce BCA Protein Assay Kit (Thermo Scientific, Rockford, IL, USA). All the results were expressed as ratios relative to total protein content (pg/mg protein).

### 2.5. Behavioral and Locomotor Assessment

The experimental animals used for behavioral tests were maintained under standard conditions of temperature and humidity and received water and food ad libitum until adulthood. After PND 60, male rats randomly selected from each experimental group (7 pups/group) were transferred to the neurobehavioral testing laboratory in their home cages. Only male animals were selected for behavioral testing to avoid data variability that might be caused by the estrous cycle in female rats [19,20]. The tests were performed in the second half of the day considering the rat-specific circadian rhythm.

The novel object recognition test (NORT) consisted of two trials: an accommodation trial in which the rat explored for 10 min two identical objects (sample objects) followed by a retention period of either 10 min or 24 h, depending on the type of memory being explored (short or long-term memory). After the initial trial, the rat was exposed to a testing trial of another 10 min consisting of a presentation with a sample object and a new object. Every rat underwent the test sessions for both short and long-term memory. Between these two test trials, the novel object was replaced. The discrimination index (DI) was calculated based on the total time spent exploring the new and familiar object using the formula: DI = (tn − tf)/(tn + tf), where tn represents the time spent exploring the new object and tf represents the time spent exploring the familiar object. The NORT is a commonly-used memory test and provides a direct readout of hippocampal-dependent recognition memory [21]. Rats are naturally explorative animals and therefore should have a preference for the novel over the familiar object. The test is based on spontaneous behavior: the main assumption is that access to novelty (e.g., an object or an environment) can elicit approach behaviors in animals with intact hippocampal function. This apparent unconditioned preference for novelty is the principle of this test because they can maintain a representation of the familiar objects stored in memory. The rat preference in the testing trial to the sample object corresponded to altered memory. All these issues made NORT quick and simple to be implemented and, therefore, it had been widely used for assessing mild cognitive impairment in pre-clinical research [22,23].

The Forced Swimming Test (FST) was performed in a transparent Plexiglas cylinder (20 cm in diameter and 40 cm high) from which the animals could not escape. The accommodation trial consisted in placing the rat in the cylinder containing water at 25 °C at a depth of 6 cm for 7 min. After 24 h, the rats were tested by placing them in the same cylinder containing water at a depth of 18 cm for 5 min, in order not to touch the bottom of the cylinder with the posterior legs [16]. The FST is based on the evaluation of rats exposed to water. They initially express an escape-directed behavior, such as swimming and climbing, and afterward they stop struggling, showing passive immobile behavior. The initial pre-test trial is required because it induces a faster immobility state in rats during the test session and a more accurate analysis [24]. FST is one of the most commonly used tests for studying depressive-like behavior in rodents. It is a form of inducing “behavioral despair”, which means that the animal loses hope to escape the stressful environment, which can be quantified using the immobility time. This time frame is considered a behavioral marker of exhaustion and depression [25,26].

### 2.6. Statistical Analysis

The statistical analysis was performed using SPSS version 20.0 and graphical representations were done using GraphPad Prism 6.00 (GraphPad Software Inc., California). For ELISA, the data were expressed in pg/mg total protein for each probe.

Regarding behavioral tests, in FST, the immobility time was expressed in minutes. In NORT, the discriminative index varied between −1 and +1. The tendency to explore the familiar object was revealed by a negative index and vice versa.

The NORT, FST and hippocampal S-100B and IL-1B (ELISA) data were compared using one-way measure ANOVA followed by posthoc Tukey’s test, assuming a normal distribution. Continuous data from all experiments were expressed as mean ± SEM. A two-sided *p*-value < 0.05 was considered statistically significant.

## 3. Results

### 3.1. Hippocampal Concentration of S-100B and IL-1B

**Figure 2:**
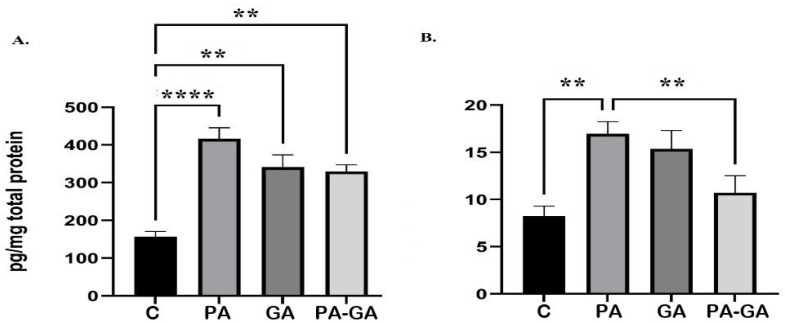
Hippocampal S-100B protein (**A**) and interleukin IL-1B (**B**) assessed in 15-day-old Wistar rats exposed to perinatal asphyxia (PA) and/or anesthesia (GA): C-control group, PA-perinatal asphyxia group, GA-anesthesia group, and PA-GA-perinatal asphyxia and anesthesia group. Bars are means ± SEM. ** represents *p*-value between 0.01–0.001, and **** represents *p*-value ˂ 0.0001.

The result revealing the level of hippocampal S-100B protein among groups is illustrated in Figure 2A. Comparing with the C group, we observed a statistically significant increase of hippocampal S-100B levels in all other groups: PA vs. C (441.85 ± 37.06 vs. 157.47 ± 13.2, p˂0.0001), GA vs. C (341.09 ± 32.8 vs. 157.47 ± 13.2, *p* = 0.005) and PA-GA vs. C (329.88 ± 17.49 vs. 157.47 ± 13.2, *p* = 0.007). No statistical variation was observed between the means of PA and PA-GA (441.85 ± 37.06 vs. 329.88 ± 17.49, *p* = NS).

The concentration of IL-1B in the four groups is shown in Figure 2B. We identified a statistically significant increase of hippocampal IL-1B in the PA group compared with the C group (16.95 ± 1.3 vs. 8.26 ± 1.05, *p* = 0.01) and a statistically significant decrease in PA-GA group comparing with PA group (10.7 ± 1.82 vs. 16.95 ± 1.3, *p* = 0.04). The concentration of hippocampal IL-1B in all other groups compared with each other revealed no statistically significant difference: GA vs. C (15.38 ± 1.92 vs. 8.26 ± 1.05, *p* = NS) and PA-GA vs. C (10.7 ± 1.82 vs. 8.26 ± 1.05, *p* = NS).

### 3.2. Behavioral and Locomotor Assessment

**Figure 3:**
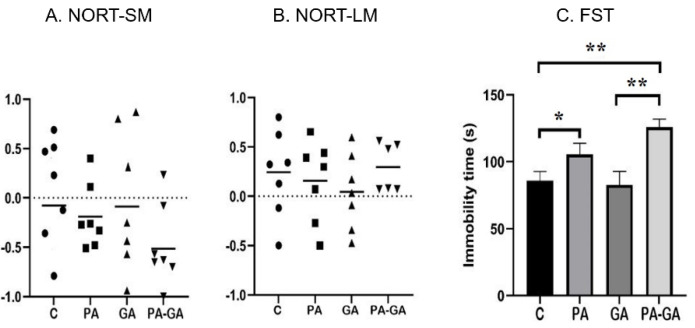
**Behavioral and locomotor assessment**. The discriminative indices in NORT for short- and long-term memory (SM and LM) respectively (**A**,**B**) and immobility time in FST (**C**). The assessment was performed at maturity (after P60) in male rats exposed to perinatal asphyxia (PA) and/or anesthesia (GA): C-control group, PA-perinatal asphyxia group, GA-anesthesia group, and PA-GA-perinatal asphyxia and anesthesia group. Bars are means ± SEM. * represents *p*-value between 0.01–0.05 and ** represents *p*-value between 0.001–0.01.

The results for the behavioral assessment are illustrated in Figure 3. In NORT the discriminative indexes assessing the short-term memory are comparable among groups: C (−0.07 ± 0.12), PA (−0.191 ± 0.12), GA (−0.08 ± 0.3), PA-GA (−0.51 ± 0.13) (Figure 3A). We observed similar results in NORT trials assessing the long-term memory: C (0.24 ± 0.19), PA (0.15 ± 0.16), GA (0.04 ± 0.14), PA-GA (0.29 ± 0.1) (Figure 3B).

In FST the immobility time was expressed in seconds, and we observed a statistically significant difference between C and PA groups (85.8 ± 6.88 vs. 105.5 ± 8.27, *p* = 0.03), C and PA-GA groups (85.8 ± 6.88 vs. 125.8 ± 5.97, *p* = 0.01), and GA and PA-GA groups (82.67 ± 10.18 vs. 125.8 ± 5.97, *p* = 0.007) (Figure 3C).

## 4. Discussion

Among the most important healthcare concerns in the contemporary medical world are the deleterious consequences of PA. Severe reduction in blood oxygenation and blood supply to the fetal brain during the vulnerable stage of neurodevelopment, may lead to hypoxic-ischemic encephalopathy and subsequent life-long neurological disorders, such as mental impairment and cerebral palsy [27].

The recommendations would be to titrate the anesthesia dose, to counteract the synergic nociceptor perception and stimulation and to offer a stress-free environment for the children at anesthesia induction.

One difference between the mature and the immature brain is that the latter has a more active immune system formed by amoeboid microglial cells, with roles in neurodevelopment. When they become activated by inflammation, microglial cells switch to a pro-inflammatory state and increase in number. They attract peripheral immune cells to the central nervous system by producing and releasing numerous inflammatory mediators such as IL-1B, IL-6, and tumor necrosis factor α [28,29]. IL-1B, a leading pro-inflammatory cytokine, represents a crucial step of the neuroinflammatory cascade. It displays pleiotropic roles in the induction of central inflammatory processes by release of other cytokines and mediators. It also modulates and amplifies the sickness response and the subsequent physiological and behavioral impairments [30]. Our results confirmed the hypothesis that PA induces neuroinflammation by increasing the level of hippocampal IL-1B. Also, we observed that inhalation anesthesia on a non-exposed immature brain does not appear to induce neuroinflammation. Although the neuroinflammation level resulting from the double exposure to PA and anesthesia during childhood is lower compared to PA alone, this result could reveal an auto-limited and reversible neuroinflammatory process secondary to PA. Also, a possible mechanism could be a later peak or cytokines level. Our results are in accordance with Shen et al. [31], who observed that a single anesthesia exposure to sevoflurane does not increase the neuroinflammation in the brain of young mice. Further issues regarding multiple anesthesia exposures or the effect of concurrent surgical stress on the immature brain should be, however, addressed in the future.

In the presence of an intense neuroinflammatory process, the S-100B protein, a calcium-binding peptide [32], is a reliable indicator to measure the extent of brain damage through glial activation and/or death when is released into the circulation from its major sources, astroglial and Schwann cells [33]. These glial cells closely interact with microglia and the inflammatory factors release a myelin formation in the brain, thus modulating the response to various brain injuries [28]. In small amounts, due to paracrine and autocrine effects on neurons and glia, S-100B plays an important role in neurodevelopment and recovery after injury [34]. Our quantitative results for S-100B have a similar pattern to that observed when analyzing IL-1B. We have shown that PA is accompanied by an increased hippocampal S100B level compared to normoxia, probably as a result of reactive astrogliosis [35]. Moreover, we observed that inhalation anesthesia produced a comparable increase in the hippocampal S-100B. Thus, the process of glial activation secondary to inhalation anesthesia, in the absence of neuroinflammation, could represent a potential adaptive mechanism of the young brain regardless of the previous PA exposure. No cumulative effect of inhalation anesthesia and PA in the immature brain was documented. Due to the paucity of data in the literature, no further analysis could be done to address the plausibility of our result. Further consistent research in this area should be conducted to confirm this hypothesis.

To assess the cognitive and behavioral impairments secondary to PA and inhalation anesthesia, we performed two behavioral tests: NORT and FST.

Our study showed no alteration of the short-term memory among groups as assessed by the NORT. Conversely, Zhou et al. demonstrated using a general anesthesia model in Sprague-Dawley neonatal rats, that anesthesia impairs short-term memory [36]. Although the anesthesia exposure in our study was not performed at the same age as in the study mentioned above, awareness should be raised in this particular research area, by designing further experimental studies to identify the age of maximal brain vulnerability. Similarly, regarding long-term memory, no significant differences between the groups could be mentioned. Simola et al. identified long-term neurological impairments after PA in adult male rats, but there is a paucity of data concerning the impact of inhalation anesthesia on long-term memory in rats revealed by NORT [37].

Our results showed that PA impacts depressive-like behavior as assessed by the FST, by increasing the immobility time, whilst anesthesia has no influence. However, we identified an altered depressive-like behavior in male rats cumulatively exposed to both PA and inhalation anesthesia. In this context, inhalation anesthesia during childhood could impact some behavioral features in offspring previously exposed to PA. Conversely, some data from the literature advocated the antidepressant and anxiolytic effects of general anesthesia in experimental settings [38,39]. Due to the heterogeneity of the anesthesia regimen used, further clinical trials are needed to confirm this hypothesis.

### Study Limitations

As a study limitation we would mention the lack of histopathology and immunohistochemistry assessment of the hippocampal tissue, that could give the possibility to corroborate the results of the present study. Other limitation could be represented by the inbreeding although we tried to prevent this by using pups from four mothers per group. Pups were randomly distributed within groups. Additionally, considering that no pups died during PA exposure, we can assume that our exposure model leads to a less severe brain damage, which could mimic the clinical scenario for birth asphyxia. Sevoflurane was mixed in 100% O_2_ as a safety measure, without considering the potential detrimental effect of hyperoxia. Finally, the ELISA assessment was performed immediately after anesthesia exposure only. No other time points were considered. We will address this issue, however, in a future study.

## 5. Conclusions

To our best knowledge, this is the first study that investigates the role of general anesthesia on the developing brain previously exposed to PA, with outcomes considering various injury markers like hippocampal IL-1B and S-100B together with complex behavioral assessment tests in Wistar rats. Our results support the detrimental effects of PA on neuroinflammation, glial activation, and behavioral changes. Moreover, a single exposure to inhalation anesthesia of the developing animal brain during childhood produces a self-limited glial activation, revealed by hippocampal S-100B level and minor behavioral impairments like lucrative memory, depressive-like behavior, and locomotion. No cumulative consequences of inhalation anesthesia on the immature brain previously exposed to PA were observed, but further studies are needed to better assess the immature brain response to these exposures and to rise the clinician’s awareness of the potential risks of cumulative exposure.

In conclusion, our study sets the stage for future extensive research in the field of pediatric anesthesia and neonatal intensive care, defining new risk factors for modern therapeutic conduct, preventing abnormal behaviors and psychiatric conditions, and, ultimately, improving quality of life.

## Data Availability

Not applicable.
